# Long-term cigarette smoke exposure dysregulates pulmonary T cell response and IFN-γ protection to influenza virus in mouse

**DOI:** 10.1186/s12931-021-01713-z

**Published:** 2021-04-20

**Authors:** Wenxin Wu, Lili Tian, Wei Zhang, J. Leland Booth, Erola Ainsua-Enrich, Susan Kovats, Brent R. Brown, Jordan P. Metcalf

**Affiliations:** 1 Pulmonary, Critical Care and Sleep Medicine, Department of Medicine, University of Oklahoma Health Sciences Center, Room 425, RP1, 800 N. Research Pkwy., Oklahoma City, OK 73104 USA; 2 Arthritis and Clinical Immunology Program, Oklahoma Medical Research Foundation, Oklahoma City, OK 73104 USA; 3 Department of Microbiology and Immunology, University of Oklahoma Health Sciences Center, Oklahoma City, OK USA; 4 Veterans Affairs Medical Center, Oklahoma City, OK USA

**Keywords:** Cigarette smoke, Influenza, Virus, T lymphocyte, IFN-γ, CTL

## Abstract

**Background:**

Influenza is a highly contagious, acute, febrile respiratory infection caused by a negative-sense, single-stranded RNA virus, which belongs in the Orthomyxoviridae family. Cigarette smoke (CS) exposure worsens influenza infection in terms of frequency and severity in both human and animal models.

**Methods:**

C57BL/6 mice with or without CS exposure for 6 weeks were inoculated intranasally with a single, non-lethal dose of the influenza A virus (IAV) A/Puerto Rico/8/1934 (PR8) strain. At 7 and 10 days after infection, lung and mediastinal lymph nodes (MLN) cells were collected to determine the numbers of total CD4 + and CD8 + T cells, and IAV-specific CD4 + and CD8 + T cells, using flow cytometry. Bronchoalveolar lavage fluid (BALF) was also collected to determine IFN-γ levels and total protein concentration.

**Results:**

Although long-term CS exposure suppressed early pulmonary IAV-antigen specific CD8 + and CD4 + T cell numbers and IFN-γ production in response to IAV infection on day 7 post-infection, CS enhanced numbers of these cells and IFN-γ production on day 10. The changes of total protein concentration in BALF are consistent with the changes in the IFN-γ amounts between day 7 and 10, which suggested that excessive IFN-γ impaired barrier function and caused lung injury at the later stage of infection.

**Conclusions:**

Our results demonstrated that prior CS exposure caused a biphasic T cell and IFN-γ response to subsequent infection with influenza in the lung. Specifically, the number of IAV antigen-specific T cells on day 10 was greatly increased by CS exposure even though CS decreased the number of the same group of cells on day 7. The result suggested that CS affected the kinetics of the T cell response to IAV, which was suppressed at an early stage and exaggerated at a later stage. This study is the first to describe the different effect of long-term CS on T cell responses to IAV at early and late stages of infection in vivo.

**Supplementary Information:**

The online version contains supplementary material available at 10.1186/s12931-021-01713-z.

## Introduction

Cigarette smoking (CS) is a significant public health problem. It is the primary cause of chronic obstructive pulmonary disease (COPD) in developed nations, and predisposes those with COPD to severe respiratory tract infections [1]. CS exposure alone increases the frequency and severity of respiratory tract infections [2] and is associated with more frequent and more severe infections with influenza A virus (IAV) [3]. IAV, a negative-sense single strand RNA virus, is a highly contagious agent that causes upper and lower respiratory tract infection resulting in 200,000 hospitalizations and 36,000 deaths in the United States per year [4, 5]. Our previous studies in human and animal models have shown that this predisposition to IAV infection in CS individuals is due to immunosuppression of the antiviral innate response to IAV [6–8].

CD4 + and CD8 + T cells contribute to control of IAV infection. T cells recognize more conserved components, such as the nucleoprotein (NP), of the IAV that are far less likely to mutate [9, 10]. The transfer of antigen-experienced IAV-specific CD8 + and CD4 + T cells into naive recipient mice can confer protection against heterologous viruses [11, 12]. CD4 + T cells provide help to B cells, promote expansion of CD8 + cytotoxic T lymphocytes (CTL), and lyse infected cells [13]. In murine models, CD8 + CTL are believed to be the main mediators of IAV clearance, and were essential in reducing viral titers and protecting against lethal viral challenge through cytolysis of the virus-infected target cells and production of IFN-γ that further enhanced antiviral inflammation [14–16]. CD8 + T cell depleted mice have delayed viral clearance and increased mortality after IAV challenge [17, 18]. IFN-γ activates immune cells, upregulates immunomodulating molecules, and modulates antibody isotype switching. IFN-γ is produced predominantly by natural killer (NK) cells first as part of the innate immune response, and by Th1 CD4 + and CD8 + CTL effector T cells once antigen-specific immunity develops [19]. While other cell types produce IFN-γ, peak IFN-γ production coincides with the arrival of IAV-specific CD4 + cells and CD8 + CTLs into the respiratory tract, and acute removal of both subsets with depleting antibodies effectively eliminates detectable IFN-γ [20].

IAV infection of the airways induces the maturation of respiratory dendritic cells (DCs), which migrate from the lung to the mediastinal lymph nodes (MLNs) carrying IAV antigens, leading to initiation of an IAV-specific T cell response [21]. Naïve T cells preferentially migrate through secondary lymphoid organs such as lymph node and spleen where they encounter DCs [22, 23]. After processing the protein antigens of the IAV into short peptides, DCs display these peptides on their surface. During the 1–3 days within the draining lymph node, the CD8 + T cells receive signals through their TCR and costimulatory molecules such as CD28. These signals drive proliferation and differentiation of the naive CD8 + T cells into activated effector T cells and memory cells [24]. CD8 + T cells then migrate through the space between the blood vessel and airway epithelium and cross the basement membrane of the epithelium to reach the infected airway epithelial cells [25]. The exact effects of CS on DC maturation and T cell activation is still a matter of debate, with human, animal, and in vitro investigations yielding conflicting results [26–29].

We have shown that CS exposure increases mortality from IAV infection [30]. However, the role of CS-impaired T cell function during IAV infection is not clear, and is the subject of this study. Prior reports showed that IFN-γ cytokine release and proliferation were attenuated in T cells from CS-exposed mice up to day 7 after IAV infection [31, 32]. Our previous study also found that CS treatment suppressed IAV-stimulated IFN-γ protein induction in bronchoalveolar lavage fluid (BALF) from the lung at day 7 after infection [7]. However, it is well established that the number of NP-specific CD8 + T cells peaks at 10–12 day after infection in the MLNs and lungs [33]. Thus, it is important to compare the effect of CS on the generation of IAV-specific T effector cells and IFN-γ production on day 7 and 10 after IAV infection. We will focus on the CS effects on the kinetics of T cell responses in this report.

## Materials and methods

### Preparation of influenza virus stock and plaque assays

H1N1 influenza virus, A/PR/34/8 (PR8), was passaged in Madin–Darby canine kidney (MDCK) cells. Virus was grown in MDCK cells in DMEM/F12 with ITS + (BD Biosciences, Franklin Lakes, NJ) and trypsin, harvested at 72 h post-infection, and titered by plaque assay in MDCK cells. There was no detectable endotoxin in the final viral preparations used in the experiments as determined by limulus amebocyte lysate assay (Cambrex, Walkersville, MD). The lower limit of detection of this assay is 0.1 EU/ml or approximately 20 pg/ml LPS. For determination of viral titers in infected mice, whole mouse lungs were collected and homogenized in 1 ml of ice cold PBS. Solid debris was pelleted by centrifugation and viral titer was determined using a standard plaque assay on MDCK cells [7]. Results were expressed as PFU/ml of extract.

### Animals

C57BL/6 mice with were bred under pathogen-free conditions in the animal facility at the Oklahoma University Health Sciences Center. Mice were housed at 20 °C on a 12 h light/dark cycle in sterile microisolator cages and fed ad libitum with sterile chow and water. We used both males and females for our experiment. The Institutional Animal Care and Use Committee of the Oklahoma University Health Sciences Center approved all of the animal protocols.

### Whole-body CS exposure

Whole-body CS exposure was performed as described [7]. Mice, starting at 6 weeks of age, were exposed to the smoke of 3R4F reference cigarettes (University of Kentucky, Lexington, KY) for 5 h per day. Mice receiving CS were gradually brought up to the target exposure over a period of 2 weeks, and treated 5 days/week for 6 weeks. Treatment was administered by placing mice in a Plexiglas smoking chamber (Teague Enterprises, Davis, CA). Smoke exposure was standardized to total suspended particles = 90 mg/m^3^, 11% mainstream and 89% sidestream smoke in the chamber of the machine. “Nonsmoking” (NS) treatment groups were conducted for the same periods of time, but mice were exposed to filtered room air.

### Influenza virus infection

IAV infection was performed under isoflurane anesthesia. IAV PR8 stock was diluted in PBS to make non-lethal dose of the virus (300 PFU/mouse). The mice were infected with IAV immediately after the last CS exposure. The virus solutions (50 µl) were administered by intranasal instillation as the animal was held in a vertical position. Control animals received an equal volume of PBS.

### Bronchoalveolar lavage (BAL)

Mice were sacrificed using isofluorane. BAL was performed using a closed thorax technique by exposing the trachea, nicking the bottom of the larynx and inserting a 3/4-inch 22-gauge cannula into the proximal trachea. The proximal end of the trachea was tied off, and 0.6 ml of sterile PBS was gently introduced into the lungs and recovered. This was repeated 3 times for a total instilled volume of 1.8 ml. Return volume varied by < 10% between samples. BAL fluid (BALF) was centrifuged to remove cells and was stored at − 20 °C. Total protein in BALF was determined by a Pierce BCA Protein Assay Kit (ThermoFisher Scientific, Waltham, MA).

### Cytokine assays

IFN-γ, CXCL10 and Granzyme B in the BALF was measured using ELISA (R&D systems, Minneapolis, MN).

### Measurement of mRNA expression by quantitative real-time PCR (qRT-PCR)

Total RNA from lung was extracted using a modified TRIzol (Invitrogen, Carlsbad, CA) protocol and spectrophometrically quantitated. The integrity of RNA was verified by formaldehyde agarose gel electrophoresis. Equal amounts (1 µg) of RNA from each sample were reverse-transcribed into cDNA with the oligo (dT) SuperScript II First-Strand Synthesis System for RT-PCR (Invitrogen). qRT-PCR was performed using 100 ng sample RNA and SYBR Green (Quanta Biosciences, Gaithersburg, MD) in a Bio-Rad CFX96™ Touch Real-Time PCR Detection System. Results were calculated and graphed using the ΔCT of the target gene and normalizer, β-actin. The primers’ sequences were as follows: IFN-γ forward 5′- GGTCATTCAGATGTAGCGG-3′; IFN-γ reverse 5′- CACTCTCCTCTTTCCAATTC-3′; β-actin forward 5′- GCCAACCGCGAGAAGATGACC-3′; β-actin reverse 5′- CTCCTTAATGTCACGCACGATTTC-3′; CXCL9 forward 5′-TGTGGAGTTCGAGGAACC CT-3′; CXCL9 reverse, 5′-TGCCTTGGCTGGTGCTG-3′; Granzyme B forward 5′-TGTTTTCTCTGCCATCTGCTCTC; Granzyme B reverse 5′- GCTTTGTAAAAGTCTCCAGCCTGTG-3′; CXCL10 forward 5′-GGTCCGCTGCAACTGCATCC-3′; CXCL10 reverse 5′- GCAATTAGGACTAGCCATCC-3′; IAV M1 Protein forward 5′- ATGAGCCTTCTAACCGAGGTC-3′; IAV M1 Protein reverse 5′- TGGACAAAACGTCTACGCTGCAG-3′.

### Isolation of cells from lung tissue [34]

Lungs were perfused with PBS + 1 mM EDTA before digestion for 60 min with Liberase (0.1 mg/ml) and DNase I (0.1 mg/ml) (all from Roche) in PBS + 0.5% BSA pH 7.4. Mediastinal lymph nodes (MLN) were mechanically disrupted into single-cell suspensions. Lung and LN cells were filtered (70 and 40 μM, respectively), washed with RPMI + 10% FCS or HBSS without Ca2 + and Mg2 + , respectively, and red cells lysed using RBC lysis buffer (BD Biosciences).

### Ex vivo T cell stimulation

To determine the numbers of CD4 + and CD8 + T cells producing IFN-γ ex vivo, 3 × 10^6^ isolated cells were incubated with the H-2D^b^-binding NP366-374 peptide (2 μg/ml) (MBLI, Woburn, MA) and the I-A^b^-binding NP311-325 peptide (2 μg/ml) (Bio-Synthesis Inc, Lewisville, TX) and brefeldin A (5 μg/ml) (BD Biosciences) in RPMI + 5%FCS for 5 h. Surface and intracellular staining identified IFN-γ producing CD4 + and CD8 + T cells.

### Flow cytometry

For surface staining, cells were incubated with monoclonal antibodies (mAbs) on ice for 15 min after 5 min of anti-CD16/32 treatment in FACS buffer (PBS, 5% newborn calf serum, 0.1% NaN3). Kits for intranuclear staining (ThermoFisher Scientific) and intracellular cytokine staining (BD Biosciences) were used according to the manufacturer’s instructions. Lung and MLN T cells were identified with a fluorochrome-linked mAb cocktail, including CD45.2-APCCy7, IFN-γ-PE, CD3-PECy7, CD4-BV786 and CD8-APC. mAbs were purchased from BD Biosciences, Biolegend, Tonbo Biosciences, and ThermoFisher Scientific. Cells from lung were stained for flow cytometry to determine the numbers of CD8 + T cells binding H-2Db/NP366-374 tetramers and numbers of CD4 + T cells binding I-Ab/NP311-325 tetramers using MHC I or MHC II-tetramers containing viral NP peptides to identify IAV antigen-specific CD4 + and CD8 + T cells in vivo. Tetramers of H-2D^b^-NP366-374-PE were purchased from MBL International. Tetramers of I-A^b^-NP311-325-APC and negative control I-A^b^-PVSKMRMATPLLMQA-APC were obtained from the NIH Tetramer Core Facility. Live/dead cell discrimination was done with a fixable Zombie Aqua™ dye (Biolegend, San Diego, CA). Samples were acquired on an LSRII instrument containing four lasers (BD Biosciences) and analyzed using FlowJo software (Treestar, Ashland, OR).

### Statistical analysis

Where applicable, the data were expressed as the mean ± standard error of the mean (SEM). Statistical significance was determined by one-way ANOVA with Student–Newman–Keuls post hoc correction for multiple comparisons. Significance was considered as p < 0.05.

## Results

### CS exposure decreased IAV antigen-specific CD4 + and CD8 + IFN-γ + T cells in the lung on day 7 but increased the same cells on day 10 post-infection (p.i.)

**Fig. 1:**
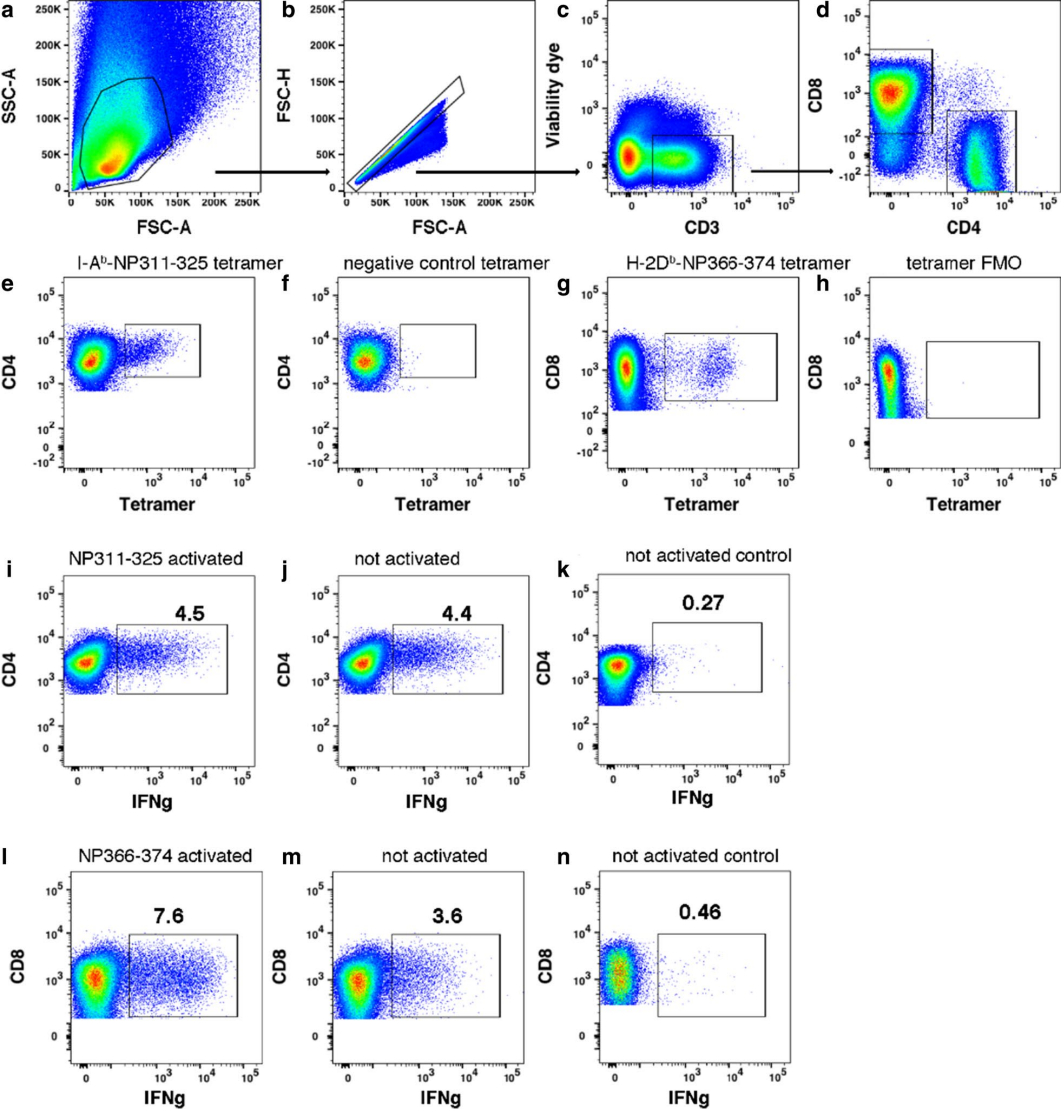
Gating of influenza antigen-specific T cells in the lung. **a**–**d** Sequential gating of lymphocytes, singlets, viable CD3 + T cells, and CD4 + and CD8 + T cells on day 7 p.i. **e**, **f** Gating of CD4 + T cells binding the I-A^b^-NP311-325 tetramer or the negative control I-A^b^-PVSKMRMATPLLMQA tetramer on day 7 p.i. **g**, **h** Gating of CD8 + T cells binding the H-2Db-NP366-374 tetramer or the tetramer FMO control on day 7 p.i. **i**, **j** Gating of IFN-γ + CD4 + T cells activated with the I-Ab-binding NP311-325 peptide for 5 h ex vivo or left unstimulated on day 7 p.i. Note that peptide does not augment the production of IFN-γ because the T cells remain activated in vivo at this time point. **k** From a poor responder mouse, gating of CD4 + T cells left unstimulated for 5 h ex vivo shows few IFN-γ + cells, which serves as a negative control for anti-IFN-γ Ab staining. **l**, **m** Gating of IFN-γ + CD8 + T cells activated with the H-2Db-binding NP366-374 peptide 5 h ex vivo or left unstimulated on day 7 p.i. **n** From a poor responder mouse, gating of CD8 + T cells left unstimulated for 5 h ex vivo shows few IFN-γ + cells, which serves as a negative control for anti-IFN-γ Ab staining. The examples shown are from non-smoking mice

**Fig. 2:**
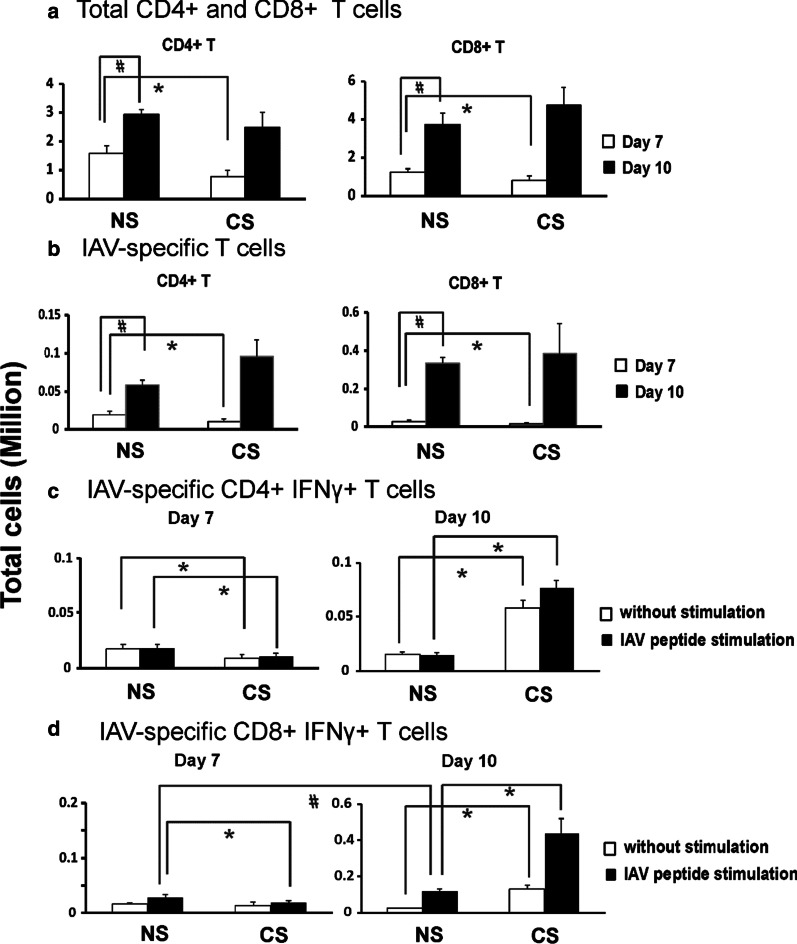
CS altered total CD4 + and CD8 + T cells and IAV-specific CD4 + and CD8 + T cells in the lung of IAV-infected mice. C57BL/6 mice were exposed to CS (CS) or not (NS) for 6 weeks in a smoke exposure chamber (Teague Enterprises), then CS-exposed mice and NS mice were intranasally inoculated with a sublethal dose of IAV (300 pfu/mouse). At 7 and 10 days post IAV infection, lung cells were isolated and stained for flow cytometry to determine the total CD4 + and CD8 + cell numbers (**a**). **b** In vivo determination of the numbers of CD8 + T cells binding H-2Db/NP366-374 tetramers and numbers of CD4 + T cells binding I-Ab/NP311-325 tetramers in the lung on days 7 and 10. Cells from lung were stained for flow cytometry to determine the numbers of CD8 + T cells binding H-2Db/NP366-374 tetramers and numbers of CD4 + T cells binding I-Ab/NP311-325 tetramers to identify IAV antigen-specific CD4 + and CD8 + T cells. **c**, **d** Ex vivo determination of the numbers of CD4 + and CD8 + T cells producing IFN-γ after incubation of lung cells with or without NP peptide. **c** CD4 + T cells producing IFN-γ after incubation of lung cells with or without NP311-325 peptide. **d** Numbers of CD8 + T cells producing IFN-γ after incubation of lung cells with or without NP366-374 peptide. Lung cells were isolated and activated ex vivo with IAV-specific peptides: I-Ab-binding NP311-325 and H-2Db-binding NP366-374, respectively. Bar graph represents mean ± SEM (n = 5). # denotes a significant difference for NS groups between day 7 and 10 (P < 0.05, by one-way ANOVA with Student–Newman–Keuls test). * denotes a significant difference between CS and NS groups at the same time point (P < 0.05)

Whole-body CS exposure was performed as described [7]. Mice receiving CS were treated 5 days/week for 6 weeks. To determine CS effects on T cell responses during IAV infection, we infected nonsmoking (NS) and CS-exposed mice intranasally with a sublethal dose of IAV A/Puerto Rico/8/1934 (PR8) virus. Lung cells were isolated on day 7 and 10 p.i. Figure 1 shows the flow cytometry gating strategies for identification of total CD4 + and CD8 + T cell and influenza antigen-specific T cells in the lung. As expected, for NS groups, total CD4 + and CD8 + T cell numbers were significantly higher on day 10 compared to day 7 (Fig. 2a). On day 7, total CD4 + and CD8 + T cell numbers were significantly less in CS groups compared to NS groups. By day 10, total CD4 + T cell numbers had increased to the same level between CS and NS groups. Importantly, total CD8 + T cell numbers in CS group appeared greater than those in the NS group though this did not reach statistical significance.

To determine if CD4 + and CD8 + IAV antigen-specific T cells were also modulated by CS during the infection, we measured numbers of CD4 + and CD8 + NP-specific T cells in the lung using H-2D^b^/NP366-374 and I-A^b^/NP311-325 IAV tetramers on days 7 and 10. Cells from lung were stained for flow cytometry using MHC I or MHC II-tetramers containing viral NP peptides to identify IAV antigen-specific CD4 + and CD8 + T cells. Again, for the NS groups, total NP tetramer-positive (NP Tet +) CD4 + and CD8 + T cell numbers were significantly higher on day 10 compared to day 7 (Fig. 2b). Then we compared the NS versus CS groups on day 7 and 10, respectively. Although the numbers of NP tetramer-positive NP Tet + CD4 + and CD8 + T cells were significantly reduced in CS mice as compared to NS mice on day 7, the numbers of these cells in CS mice increased to similar numbers in NS mice by day 10 (Fig. 2b). To determine if antigen-specific T cells in CS and NS mice differed in functional capacity, we used an ex vivo CD8 + and CD4 + T cell re-stimulation assay with intracellular staining for IFN-γ to determine IAV-specific IFN-γ-producing T cell numbers from the mice. Specifically, lung cells were isolated on days 7 and 10 p.i. and activated ex vivo with IAV-specific peptides: I-A^b^-binding NP311-325 and H-2D^b^-binding NP366-374 for 5 h at 37 °C in RPMI + 5% FCS in the presence of Brefeldin A (Fig. 2c, d). On day 7 p.i., CD4 + IFN-γ + and CD8 + IFN-γ + T cells were both lower in CS mice as compared to NS mice (Fig. 2c, d). Since the antigen-specific T cells in the lungs were likely already activated, it was difficult to increase their activation by addition of MHC-binding peptides, especially the CD4 + T cells (Fig. 1i–l). However, the numbers of CD4 + IFN-γ + and CD8 + IFN-γ + T cells in CS mice increased significantly between day 7 and 10. CS mice ended up harboring significantly greater (4 and threefold, respectively) numbers of IAV-specific CD8 + IFN-γ + T cells and CD4 + IFN-γ + T cells on day 10 than NS mice did (Fig. 2c, d). These data demonstrated that CS suppressed the activation of CD4 + and CD8 + effector T cells in the lung during IAV infection at the early stage of infection (day 7), but led to increased numbers of IAV antigen-specific CD8 + and CD4 + T cells in the lung by day 10 p.i.

**Fig. 3:**
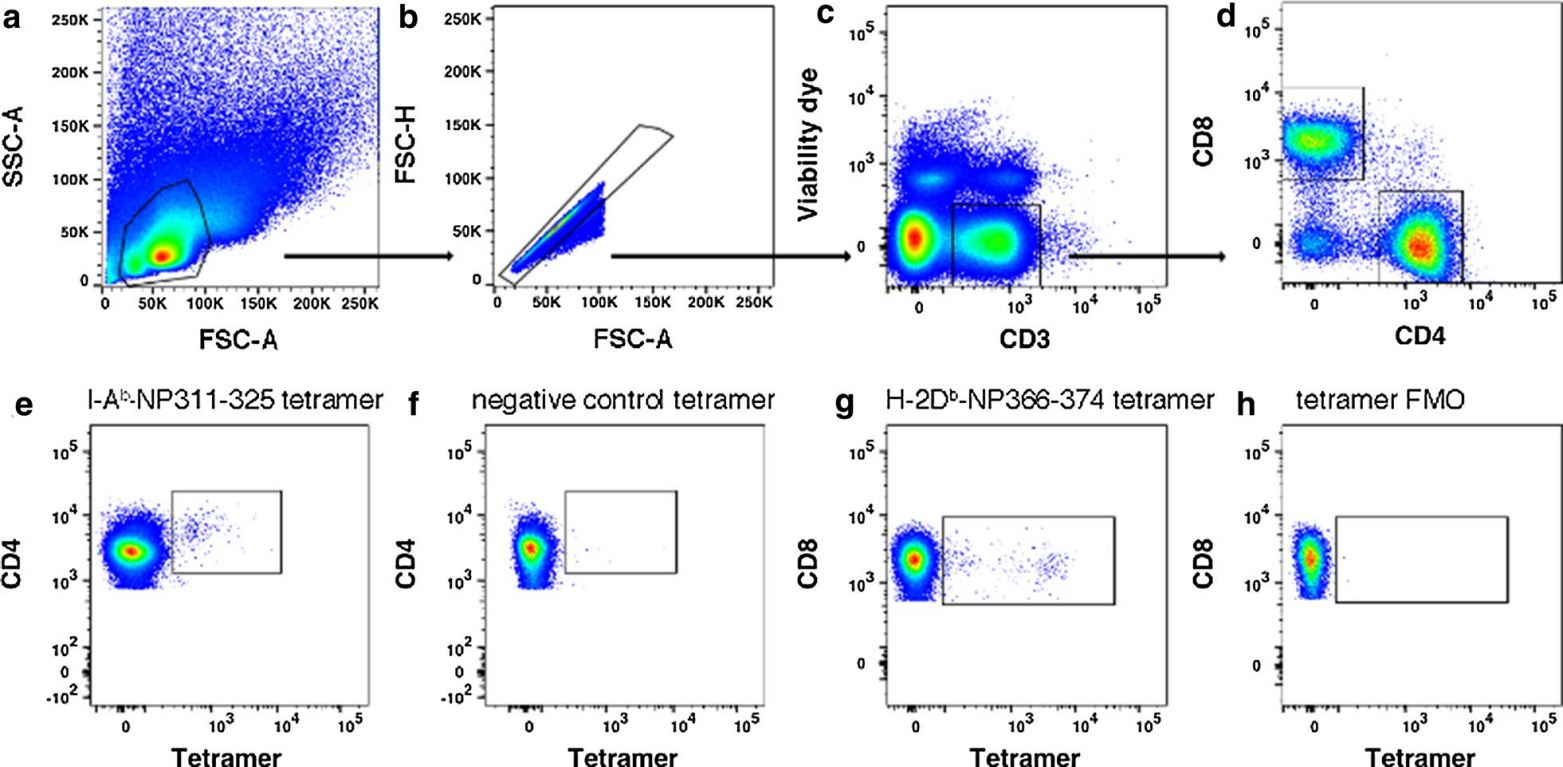
Gating of influenza antigen-specific T cells in the MLN. **a–d** Sequential gating of lymphocytes, singlets, viable CD3 + T cells, and CD4 + and CD8 + T cells. **e**, **f** Gating of CD4 + T cells binding the I-A^b^-NP311-325 tetramer or the negative control I-A^b^-PVSKMRMATPLLMQA tetramer. **g**, **h** Gating of CD8 + T cells binding the H-2D^b^-NP366-374 tetramer or the tetramer FMO control. The examples shown are from non-smoking WT mice

**Fig. 4:**
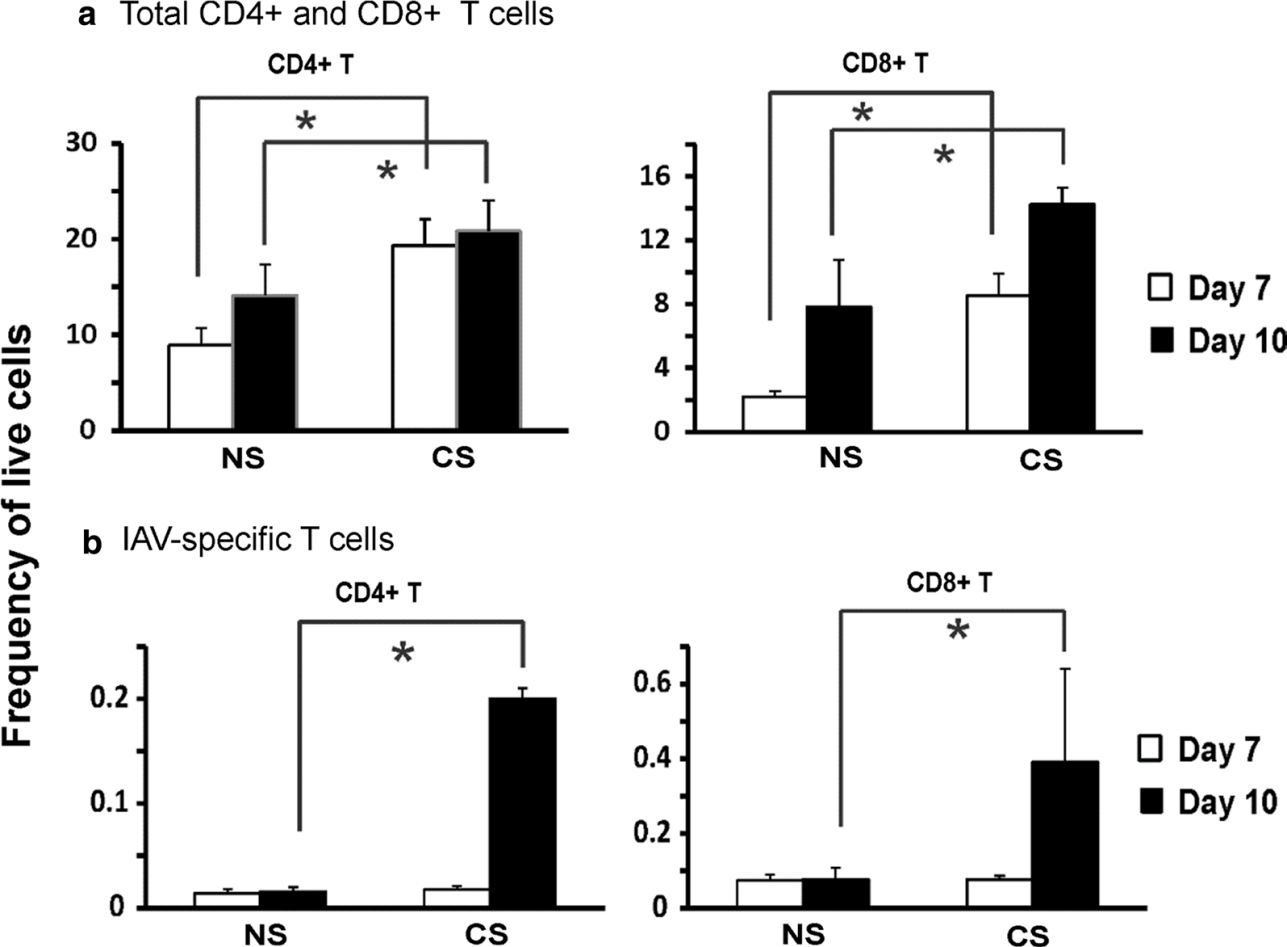
CS altered total CD4 + and CD8 + T cells and IAV-specific CD4 + and CD8 + T cells in MLN of IAV-infected mice. C57BL/6 mice were exposed to CS or not for 6 weeks in a smoke exposure chamber, then CS-exposed and NS mice were intranasally inoculated with a sublethal dose of IAV (300 pfu/mouse). At 7- and 10-days post IAV infection, MLN cells were isolated and stained for flow cytometry to determine the frequency of live cells of total CD4 + and CD8 + T cells (**a**). Cells from lung were stained for flow cytometry using MHC I or MHC II-tetramers containing viral NP peptides to identify IAV antigen-specific CD4 + and CD8 + T cells (**b**). Bar graph represents mean ± SEM (n = 5). * denotes a significant difference between CS and NS groups at the same time point

Next, we examined the total and IAV antigen-specific T cells in MLN. Cells were isolated from MLN on day 7 and 10. Since total cell numbers isolated from MLN were variable among the mice, we chose to use Frequency of Live cells (FOL) to determine the change. Live/dead cell discrimination was done with a fixable Zombie Aqua™ dye. Figure 3 shows the gating strategies of flow cytometry for identification of total CD4 + and CD8 + T cell and influenza antigen-specific T cells in MLN. FOL of total CD4 + T and CD8 + T cells in MLN of mock-infected mice are shown in Additional file 1: Fig. S1. For IAV infected mice, from day 7 to 10 in NS groups, FOL of total CD4 + T cells did not change, but FOL of total CD8 + T cells increased two fold (Fig. 4a). CS mostly increased FOL of total CD4 + and CD8 + T cells in MLN, though the difference was not statistically significant when comparing results from day 7 to day 10 (Fig. 4a). On day 7, CS did not cause any difference in FOL of IAV antigen-specific CD4 + or CD8 + T cells as judged by tetramer staining (Fig. 4b). However, the FOL of IAV antigen-specific CD4 + and CD8 + T cells in CS mice increased tenfold and fivefold, respectively, on day 10 compared to that in NS mice (Fig. 4b). Unfortunately, as there were limited cells obtained from MLN, we were unable to determine IAV-specific IFN-γ-producing T cell numbers in these tissues.

### CS exposure decreased IAV-induced IFN-γ on day 7 but increased IFN-γ on day 10 p.i in the lung

**Fig. 5:**
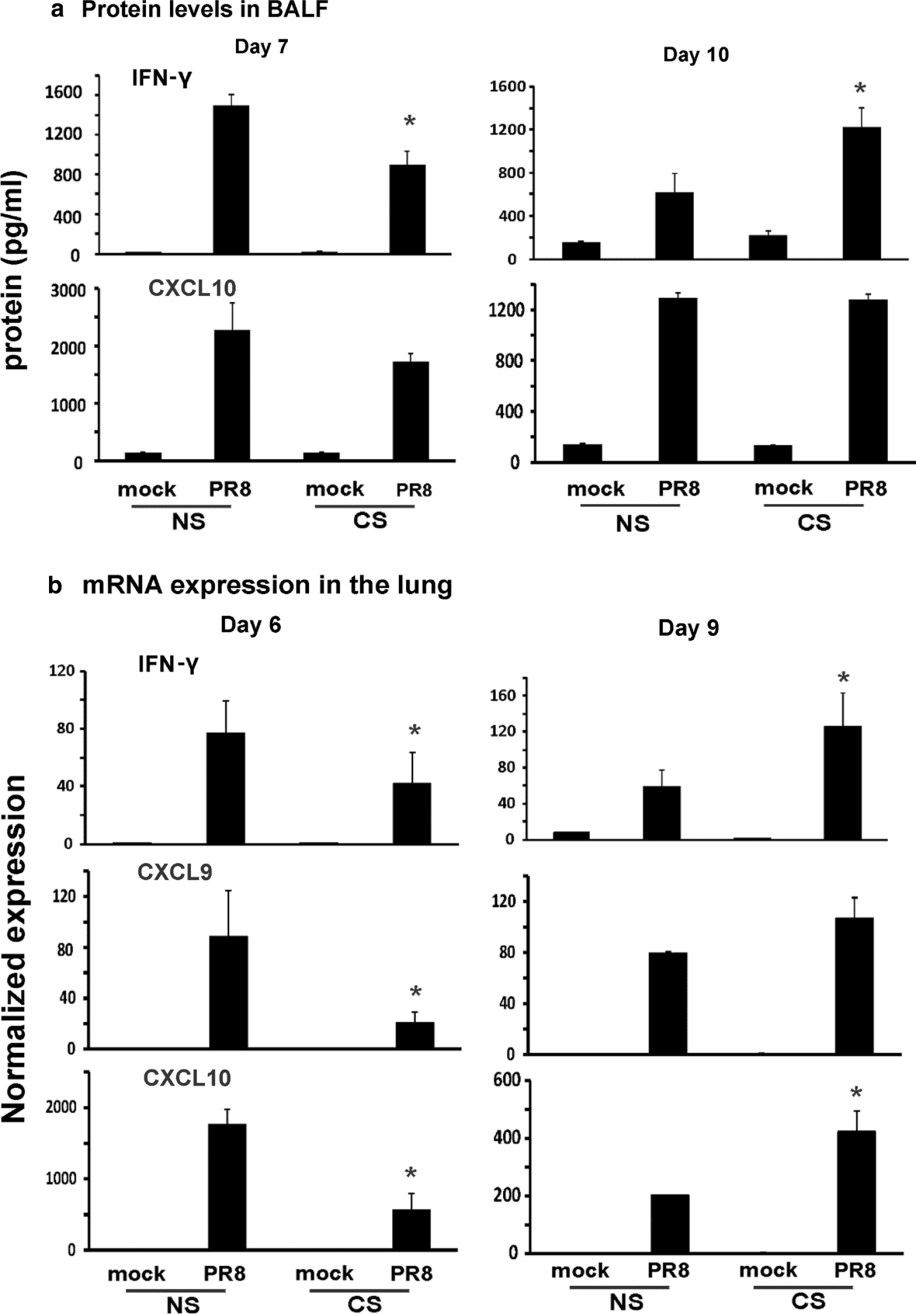
IFN-γ mRNA and protein expression were suppressed early but promoted by CS later during IAV infection. For CS groups, the mice were exposed to CS for 6 weeks in a smoke exposure chamber. Mice were infected with a non-lethal dose (300 pfu/mouse) of the IAV PR8 strain. Mock treated mice were inoculated with PBS. At the indicated time points, BALF was harvested for ELISA and lung tissues were collected for RNA preparation. **a** IFN-γ and related cytokine protein levels in BALF were determined by ELISA. **b** IFN-γ and related cytokine mRNA levels in the lung were assessed by qRT-PCR and normalized to β-actin.Data are expressed as means ± SEM. * denotes a significant difference between CS and NS groups at the same time point (P < 0.05, n = 4)

Since IFN-γ is the most prominent cytokine produced by IAV-specific T cells, we next examined IFN-γ mRNA expression in the infected lung and protein induction in BALF. Mice received CS treatment and IAV infection as previously described. BALF were collected at day 7 and 10 p.i. We found decreased IFN-γ protein levels in the BALF of CS mice compared to NS mice on day 7 as we have shown previously [7]. However, IFN-γ levels were elevated in the BALF of CS mice compared to NS mice on day 10 (Fig. 5a). This is consistent with the changes of IAV NP-specific CD8 + and CD4 + T cells capable of producing IFN-γ in the lung between day 7 and 10 in the lung. CXCL 10 protein, a chemokine downstream of IFN-γ, was decreased on day 7 in CS mice and recovered to the similar levels in NS mice on day 10 (Fig. 5a). Since mRNA expression is earlier than protein production, we selected one day earlier to assess the IFN-γ mRNA levels in the lung. We examined IFN-γ and its downstream cytokines CXCL9 and CXCL10 expression in the lung on day 6 and 9 p.i. We found decreased IFN-γ, CXCL9 and CXCL10 mRNA expression in the lung of CS mice compared to NS mice on day 6 while these cytokine mRNA expressions were significantly higher in CS mice on day 9 (Fig. 5b). Thus, IFN-γ and its downstream cytokine protein expression changes were confirmed at the transcriptional level.

### CS exposure worsened IAV-induced inflammation and epithelial cell integrity on day 10 p.i in the lung

**Fig. 6:**
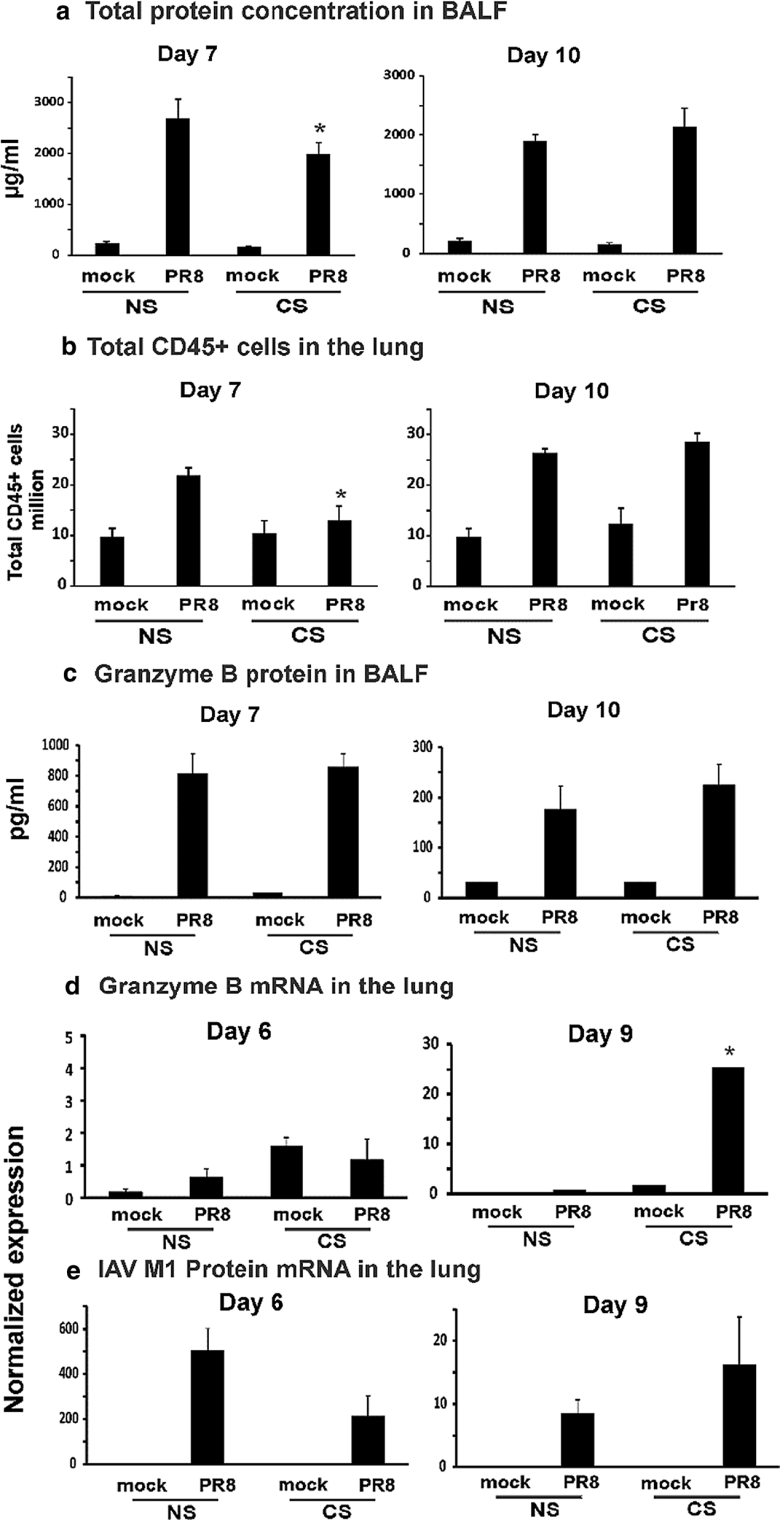
CS restrains overall inflammation in the lung at day 7 but increases inflammation at day 10 post IAV infection. For CS groups, the mice were exposed to CS for 6 weeks in a smoke exposure chamber. Then the mice were intranasally infected with a non-lethal dose of IAV PR8 (300 pfu/mouse) or mock infected with PBS. **a** Total protein concentration in BALF. BALF was harvested at the indicated time points after infection. Total protein levels in BALF were determined. Data are expressed as means ± SEM (n = 4). **b** Total CD45 + leukocytes in the lung. Lung cells were isolated and stained for flow cytometry to determine the total numbers of CD45 + cells (n = 5). **c** Granzyme B protein levels in BALF were determined by ELISA. **d** Granzyme B and **e** IAV M1 Protein mRNA levels in the lung were assessed by qRT-PCR and normalized to β-actin. Data are expressed as means ± SEM. * denotes a significant difference compared to the PR8 infected NS group at the same time point (P < 0.05)

We examined total protein concentration in BALF, an indicator of barrier integrity, which may reflect inflammatory and/or cytotoxic lung responses in mice. On day 7, total protein concentration in NS mice is significantly higher than that in CS mice (Fig. 6a). This is consistent with our earlier report which showed IAV-infected CS mice had lower total protein concentration in BALF on day 6 p.i. [30]. In contrast, total protein concentration in CS mice returned to the same level in NS mice on day 10, and appeared to surpass levels in the NS mice though this did not reach statistical significance. We also determined the total number of CD45 + leukocytes to assess the inflammatory cell infiltrate in response to IAV infection [35]. The total leukocyte number was significantly lower in CS PR8 group on day 7 p.i. The leukocyte number in CS mice also returned to the same level in NS mice on day 10 if not higher (Fig. 6b). Granzyme B is a serine protease most commonly found in cytotoxic CD8 + T cells. It is secreted by CD8 + T cells to mediate apoptosis in target cells and is involved in inducing inflammation by stimulating cytokine release. We examined this cytotoxicity/inflammation marker in our model. Granzyme B protein is at the same level in both CS and NS mice on day 7, but the protein level increased in CS mice on day 10 (Fig. 6c). Notably, Granzyme B mRNA expression in CS mouse lung was 32 fold higher than that in NS mouse lung on day 9 although the mRNA levels on day 6 were similar for both groups (Fig. 6d). Finally, we determined the viral loads by assessing IAV M1 protein mRNA expression in the mouse lung (Fig. 6e). Since we used sublethal dose (300 PFU/mouse) of virus, all animals recovered from the infection. The recovery showed in viral load changes. M1 protein mRNA expression on day 9 was much less than that on day 6 for IAV infected mice. Although M1 mRNA expression of CS group seemed to be less on day 6 and more on day 10, there was no significant difference between the NS and CS groups.

## Discussion

Exposure to CS significantly increases the risk for respiratory viral infections. CS dysregulates innate immune responses to viruses in many sophisticated and complex ways. CS primarily causes a reduced innate response in protective immune cells to IAV [36, 37]. Our previous studies have shown that CS and cigarette smoke extract (CSE) suppress antiviral innate immune responses in IAV-infected human and mouse lung, especially responses due to RIG-I [6–8]. This immunosuppressive effect of CS may play a critical role in the enhanced susceptibility of smokers to serious influenza infection. It has been demonstrated that CS exposure inhibits pulmonary T cell responses to IAV infection as well. Activated T cells produced much less IFN-γ cytokine in the lung of CS-exposed mice after IAV infection [31, 32]. However, all above experiments tracked T cell responses only up to day 7 p.i. The numbers of IAV NP-specific T cells do not peak until day 10 in the lung after infection [33]. In this report, we found, although CS exposure suppressed pulmonary total T cell responses to IAV infection on day 7, CS enhanced total T cell responses and IFN-γ production at day 10. Specifically, CS boosted IAV-antigen specific CD8 + IFN-γ + and CD4 + IFN-γ + T cells on day 10.

Gualano et al. showed that CS exposure before influenza was associated with a large increase in CD4 + and CD8 + lymphocytes and influenza-specific cytotoxic T cells in BALF at day 10 [38]. IFN-γ protein in BALF showed little change at day 3, but a rise in CS mice at day 10. However, that study used a short-term smoking model (9 cigarettes/day for 4 days before IAV infection) in BALB/c mice. Our results demonstrated that long-term smoke exposure also greatly increased both types of influenza-specific activated T cells on day 10 in the lungs of infected C57BL/6 mice. In our whole body CS exposure model, mice were exposed to the smoke of cigarettes for 4 h per day, 5 days/week for 6 weeks. The model reflects the majority of smokers, as cotinine levels in mouse blood replicate levels in typical human smokers. In addition, Gualano et al. found that CS reduced the number of activated T cells in MLNs of influenza infected mice at both day 3 and day 10. We showed here that long-term CS exposure increased IAV-specific CD4 + and CD8 + T cells in the MLNs at day 10. CS also reversed the suppression of IFN-γ released on day 7 to the extent that it greatly increased IAV NP-specific CD8 + IFN-γ + and CD4 + IFN-γ + T cells in the lung and IFN-γ production in the BALF on day 10.

IFN-γ induces apoptosis and autophagic cell death in endometrial stromal cells, bronchial and colonic epithelial cells, Hela cells, and T lymphocytes [39–43]. In the airways, IFN-γ has been implicated in virus-induced lung inflammation [44]. CD8 + T cells play a pivotal role in limiting IAV replication by lysing IAV-infected cells. However, an overexuberant CD8 + T cell response can have a deleterious effect on lung function and survival [45–47]. IAV-specific CD8 + T cells produce IFN-γ that reduce the barrier integrity of noninfected bystander epithelial cells [48]. T lymphocytes, particularly CD8 + T cells, are increased in lungs of patients with COPD [49]. Mice exposed to CS for 6 months developed emphysema, where increased CD8 + T cells and IFN-γ were blamed as the major contributors to CS-induced inflammation and emphysema [50]. We found here that the changes of total protein concentration in BALF are consistent with the lower and higher amounts of IFN-γ between day 7 and 10, which suggested that CS-induced excessive IFN-γ responses weakened barrier integrity and increased lung injury by day 10. We note that this is a non-lethal model of IAV infection, and as infection and lung injury proceeds in lethal models BAL protein levels are likely greatly increased in CS-exposed animals at later time of infection consistent with the increased mortality in this group [30]. During infection with respiratory viruses, inflammation and lung injury appears to be the primary driver of life-threatening symptoms, including infections with the recently emerged severe acute respiratory syndrome (SARS)-coronavirus (CoV)-2 [51]. The impaired viral clearance may be a less important contributor to the mortality in IAV infection [52]. Disease severity is linked to lung epithelial destruction, due to both cytopathic viral effects and immune-mediated damage. Epithelial loss contributes to acute respiratory distress syndrome, pneumonia, and increased susceptibility to bacterial superinfections [53].

## Conclusion

We found that prior CS exposure modulated the T cell response to subsequent infection with influenza. In particular, the number of IAV specific CD4 + IFN-γ + and CD8 + IFN-γ + T cells in the lung on day 10 p.i. was greatly increased by CS exposure even though the number of the same group of cells was decreased by CS on day 7. This study is the first to describe the different long-term CS effects at different stages of in vivo IAV infection on T cell responses. These results provide new insight into the mechanisms whereby CS alters the host immune system during influenza infections. It is still unknown whether the elevated T cells in CS exposed mice are from proliferation or migration from the lung tissue. The current report regarding CS effects on modulation of the T cell response to subsequent IAV infection was demonstrated in a mouse model. Parallel phenomena need to be confirmed in humans. Meanwhile, myeloid cells are rapidly recruited into the lung and play major roles in innate immunity to IAV. Further studies will be carried out to determine the source of the elevated T cells and elucidate CS effects on myeloid cells, such as neutrophils, monocytes and eosinophils, as well as on CD4 + and CD8 + T cells through the entire course of IAV infection.

## Supplementary Information

**Additional file 1: Figure S1.** Total CD4+ and CD8+ T cells in MLN of mock-infected mice.

## Data Availability

All data generated or analyzed during this study are included in this published article.

## Abbreviations

BAL: Bronchoalveolar lavage
BALF: Bronchoalveolar lavage fluid
COPD: Chronic obstructive pulmonary disease
CS: Cigarette smoking
CTL: Cytotoxic T lymphocyte
DC: Dendritic cell
FOL: Frequency of live cells
IAV: Influenza A virus
IFN: Interferon
MDCK: Madin–Darby canine kidney
MLN: Mediastinal lymph nodes
NP: Nucleoprotein
NS: Nonsmoking
PR8: A/PR/34/8
RIG-I: Retinoic acid inducible gene I
TLR: Toll-like receptor
