# Supplementary material for: Long-term cigarette smoke exposure dysregulates pulmonary T cell response and IFN-γ protection to influenza virus in mouse
Source: Respir Res. 2021 Apr 20;22:112. doi: 10.1186/s12931-021-01713-z (PMC8056367; doi:10.1186/s12931-021-01713-z)
Supplement: Supplementary file 1 — Additional file 1: Figure S1. Total CD4+ and CD8+ T cells in MLN of mock-infected mice. [file 12931_2021_1713_MOESM1_ESM.docx]

**Supplemental Fig. 1 Total CD4+ and CD8+ T cells in MLN of mock-infected mice.** C57BL/6 mice were exposed to CS or not for 6 weeks in a smoke exposure chamber, then CS exposed and NS mice were intranasally inoculated with PBS as mock-infected groups. At 7- and 10-days, MLN cells were isolated and stained for flow cytometry to determine the frequency of live cells of total CD4+ and CD8+ T cells.

Supplemental figure1
